# Supplementary material for: Validation of the Chinese Version of the Body Image Flexibility and Inflexibility Scale among Chinese College Students
Source: Behav Sci (Basel). 2024 Oct 8;14(10):910. doi: 10.3390/bs14100910 (PMC11504571; doi:10.3390/bs14100910)
Supplement: Supplementary file 1 [file behavsci-14-00910-s001.zip › behavsci-3193937-supplementary.pdf]

Table S1. The Chinese version of the BIFIS used in the study.

BIFIS	C-BIFIS
**Body image inflexibility subscale**<br>**Factor / Item**	**身体意象僵化分量表**<br>**因子/条目**
**Active avoidance**<br>1. I tried to distract myself when I felt bad about my body.<br>2. When I had a negative emotion about my body, I tried to push it away.<br>3. I tried to ignore my thoughts and feelings about my body.	**主动回避**<br>1. 当对自己身体感到不满时，我试着分散自己的注意力。<br>2. 当对自己的身体有消极情绪时，我试着回避它们。<br>3. 我试着忽视与自己身体有关的想法及感受。
**Cognitive fusion**<br>4. Negative thoughts about my body kept playing over and over in my mind.<br>5. When I had a negative emotion about my body, it was hard for me to move past it.<br>6. Negative thoughts and feelings about my body tended to stay with me for a long time.	**认知融合**<br>4. 与自己身体有关的消极想法在我脑海中反复出现。<br>5. 当对自己的身体有消极情绪时，我很难摆脱它。<br>6. 与自己身体有关的消极想法和感受往往会困扰我很长一段时间。
**Self-as-content**<br>7. I believed some of my thoughts and feelings about my body were not normal.<br>8. I thought some of my emotions about my body were bad and I shouldn't feel them.<br>9. I got upset with myself for having negative thoughts and feelings about my body.	**概念化自我**<br>7. 我认为自己对身体的一些想法和感受是不正常的。<br>8. 我觉得我对自己身体的一些情绪是不好的，我不应该感觉到它们。<br>9. 我曾因为对自己身体有消极的想法和感受而心烦意乱。
**Values disconnection**<br>10. Negative thoughts and feelings about my body distracted me from other things I care about.<br>11. When I felt bad about my body, I forgot about the	**价值脱离**<br>10. 对身体的消极想法和感受，让我很难专注于

things that matter most to me.<br>12. Negative thoughts and feelings about my body stopped me from living the life I want.	自己关心的其他事情。<br>11. 当对自己身体感到不满时，我就会忘记那些对我很重要的事情。<br>12. 对自己身体的消极想法和感受阻碍了我过想要的生活。
**Body image flexibility subscale**<br>**Factor / Item**	**身体意象灵活性分量表**<br>**因子/条目**
**Mindful acceptance**<br>13. I acknowledged my negative thoughts and feelings about my body rather than ignoring them.<br>14. I was aware of my thoughts and feelings about my body.<br>15. I was in touch with my thoughts and feelings about my body throughout the day.	**正念接纳**<br>13. 我接纳对自己身体的消极想法和感受，而不是忽视它们。<br>14. 我能意识到我对自己身体的想法和感受。<br>15. 我随时都能注意到与自己身体有关的想法和感受。
**Cognitive defusion**<br>16. I let negative emotions about my body pass without acting on them.<br>17. I let negative thoughts and feelings about my body come and go without getting stuck on them.<br>18. I was able to step back from my negative thoughts and feelings about my body.	**认知解离**<br>16. 我能让那些与自己身体有关的消极情绪过去，而不对它们采取行动。<br>17. 我能让对身体的消极想法和感受来来去去，而不陷入其中。<br>18. 我能从那些与自己身体有关的消极想法和感受中脱离出来。
**Self-as-context**<br>19. When I felt bad about my body, I tried to think about myself or the situation from a broader perspective.<br>20. I got through feeling bad about my body by thinking about the bigger picture of my life.<br>21. I saw myself as more than just my negative thoughts and feelings about my body.	**以己为景**<br>19. 当对自己身体感到不满时，我尝试从更广阔的视角来思考自己或当下的情况。<br>20. 通过从更大的生活格局去思考，我克服了对自己身体的不满情绪。

	21. 我对自己的认识不仅仅局限于对身体的消极想法和感受。
**Values connection**<br>22. I was aware of other things that mattered to me besides my thoughts and feelings about my body.<br>23. I was in touch with what is important to me, even when I had negative thoughts and feelings about my body.<br>24. Even when I had negative thoughts and feelings about my body, I still did things that were important to me.	**价值联结**<br>22. 除了对自己身体的想法和感受，我还能意识到对自己重要的其他事情。<br>23. 即使对自己的身体有消极想法和感受，我也清楚什么对自己是重要的。<br>24. 即使对自己的身体有消极想法和感受，我依然会去做那些对我很重要的事情。
